# Mechanisms underlying green flower formation

**DOI:** 10.1093/hr/uhag079

**Published:** 2026-03-03

**Authors:** Xu Li, Conghao Hong, Hao Li, Sijia Hou, Qingqing Sun, Youyi Zang, Guorun Sun, Zhimin Huang, Hongbo Gao

**Affiliations:** National Engineering Research Center of Tree Breeding and Ecological Restoration, College of Biological Sciences and Technology, Beijing Forestry University, Beijing 100083, China; State Key Laboratory of Efficient Production of Forest Resources, Beijing Forestry University, Beijing 100083, China; National Engineering Research Center of Tree Breeding and Ecological Restoration, College of Biological Sciences and Technology, Beijing Forestry University, Beijing 100083, China; State Key Laboratory of Efficient Production of Forest Resources, Beijing Forestry University, Beijing 100083, China; National Engineering Research Center of Tree Breeding and Ecological Restoration, College of Biological Sciences and Technology, Beijing Forestry University, Beijing 100083, China; State Key Laboratory of Efficient Production of Forest Resources, Beijing Forestry University, Beijing 100083, China; National Engineering Research Center of Tree Breeding and Ecological Restoration, College of Biological Sciences and Technology, Beijing Forestry University, Beijing 100083, China; State Key Laboratory of Efficient Production of Forest Resources, Beijing Forestry University, Beijing 100083, China; National Engineering Research Center of Tree Breeding and Ecological Restoration, College of Biological Sciences and Technology, Beijing Forestry University, Beijing 100083, China; State Key Laboratory of Efficient Production of Forest Resources, Beijing Forestry University, Beijing 100083, China; National Engineering Research Center of Tree Breeding and Ecological Restoration, College of Biological Sciences and Technology, Beijing Forestry University, Beijing 100083, China; State Key Laboratory of Efficient Production of Forest Resources, Beijing Forestry University, Beijing 100083, China; National Peony Gene Bank, Luoyang, Henan Province 471002, China; Luoyang Peony Industry Development Center, Luoyang, Henan Province 471002, China; National Peony Gene Bank, Luoyang, Henan Province 471002, China; Luoyang Peony Industry Development Center, Luoyang, Henan Province 471002, China; National Engineering Research Center of Tree Breeding and Ecological Restoration, College of Biological Sciences and Technology, Beijing Forestry University, Beijing 100083, China; State Key Laboratory of Efficient Production of Forest Resources, Beijing Forestry University, Beijing 100083, China

## Abstract

Green flowers are uncommon in nature, yet they present a unique opportunity to explore the molecular, developmental, and evolutionary principles underlying floral pigmentation. While most species undergo petal degreening during maturation, some retain chlorophyll through suppressed degradation, sustained synthesis, or altered plastid differentiation. Here, we synthesize recent advances in understanding the molecular basis of green flower formation, integrating evidence from plastid biology, chlorophyll metabolism, transcription factor regulation, and floral organ identity genes. Research across diverse taxa reveals that chlorophyll homeostasis in petals is shaped by the interplay of light and hormonal signals, and orchestrated by transcriptional networks. In certain instances, homeotic transformations result in leaf-like characteristics. Naturally occurring variants, as well as engineered lines, offer powerful systems to dissect how developmental programs governing organ identity intersect with pigment metabolism. Green flowers also hold distinct ornamental and cultural value, expanding their relevance beyond ecological function. By tracing progress from morphological observations to multi-omics analyses, we highlight how this field is beginning to uncover shared regulatory frameworks and lineage-specific innovations. In the future, targeted manipulation of key regulatory nodes could enable the precise breeding of stable green blooms, while comparative studies promise deeper insights into how pigment pathways evolve and integrate with broader developmental networks. Understanding these processes will not only enrich floral biology but also enhance our ability to intentionally design and diversify plant phenotypes.

## Introduction

Flower color is a crucial trait of ornamental plants, determining not only their aesthetic value but also influencing ecological interactions such as pollination. The vivid colors of most flowers primarily result from pigments, such as anthocyanins (red/purple), carotenoids (yellow/orange), and betalains (deep red/purple red). From a developmental perspective, most flower buds initially appear green due to the abundance of chloroplasts and chlorophyll within the immature petal tissues. As flowers mature, chlorophyll is degraded or chloroplasts are transformed into non-photosynthetic plastids, allowing other pigments to dominate. When flowers are fully open, chlorophyll has typically either completely disappeared or remains only in trace amounts [1]. The degreening process in flowers is believed to help flowers stand out from the leafy background when preparing to attract pollinators. However, when this typical transformation process is blocked, chloroplasts are retained and remain functional in petals, resulting in green flowers. Green flowers are very rare, constituting less than 10% of plant species worldwide (https://botany.one/2025/03/green-flowers-are-not-invisible/). In horticulture, green-flowered varieties are especially prized for their novelty and aesthetic appeal, frequently commanding high market prices. For instance, rare green mutations of ‘Dou Lv’, one of the renowned cultivars of peony, attract significant attention due to their unique coloration [2, 3]. Furthermore, the rose variety known as *Rosa chinensis* cv. ‘Viridiflora’ features all petals, stamens, and pistils transformed homotopically into green, leaf-like sepaloid structures [4]. This foliarized phenotype causes the rose to appear completely green, making it a valuable variety for studying floral organ development [4]. These observations demonstrate that, while green floral pigmentation is rare in nature, it occurs in phylogenetically diverse lineages. This prompts a fundamental question: within an evolutionary framework where chlorophyll is typically suppressed during floral organ development, through what molecular mechanisms is green floral coloration maintained?

The formation of green flowers involves multiple biological processes, including plastid development, pigment metabolic pathways and floral organ identity determination, deviating from the conventional petal maturation program. Specifically, green flowers can retain photosynthetic capacity similar to that of leaves. For instance, chloroplasts with photosynthetic activity are found in green chrysanthemum petals, and photosynthesis-related proteins, such as Rubisco, are present in carnation petals, indicating the presence of an active photosynthetic apparatus within these tissues [5, 6]. Alternatively, petals remain green because of changes in pigment metabolism. Gene expression analyses show that these green petals often have higher levels of chlorophyll biosynthesis gene expression or lower levels of chlorophyll degradation gene expression compared with normal petals [7–9]. Additionally, genetic mutations affecting floral organ identity, such as loss of function in B-class MADS-box genes, can transform petals into sepal structures that retain chlorophyll [10, 11].

Compared with the extensive research on the mechanisms underlying red and yellow flower coloration, the processes responsible for green floral coloration remain relatively obscure [12]. In this review, we summarize current research on the molecular mechanisms underlying green flower formation, with emphasis on recently identified genes and transcription factors (TFs) that promote chlorophyll accumulation or retention in green flowers, as well as genes affecting floral organ identity or development. Then, we examine the roles of chlorophyll biosynthesis and degradation within evolutionary and ecological contexts in green flowers, integrating evidence from developmental genetics, biochemistry, and physiology to outline the molecular basis of green floral pigmentation, and highlighting key knowledge gaps and priorities for future research in this underexplored field.

## Plastid development and green flower formation

Plastids are dynamic plant organelles whose versatile differentiation into specialized forms is essential for tissue and organ function and development. During early development, proplastids differentiate into specialized plastids (e.g. chloroplasts, chromoplasts, leucoplasts), which support organ formation and floral traits, such as persistent green pigmentation in petals.

### An overview of the mechanism of plastid development

**Figure 1:**
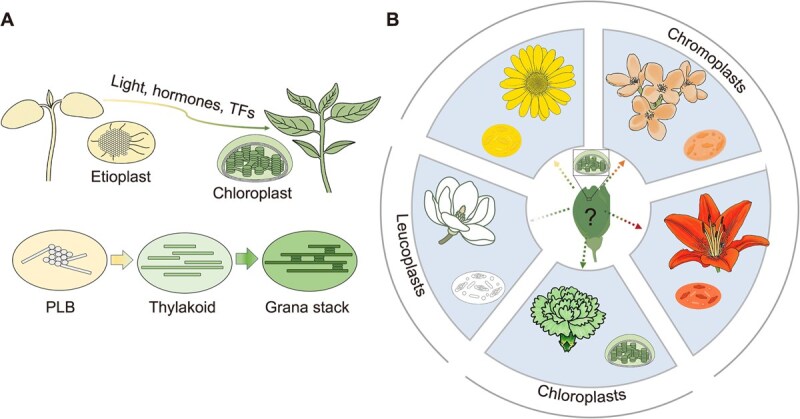
The development of plastids in leaves and petals is orchestrated by light, hormones, and key TFs. (A) Upon illumination, TFs integrate light and hormonal signals to convert etioplasts in etiolated leaves into functional chloroplasts, while the PChlide is converted into chlorophyll, driving leaf greening. During the differentiation of etioplasts into chloroplasts, the PLB gradually disaggregates and reorganizes into thylakoid lamellae, which subsequently stack to form grana. (B) Petals in closed buds contain chloroplasts. During development, specific factors can sustain these chloroplasts thus maintaining green color. Alternatively, chloroplasts may differentiate into either leucoplasts (present in white petals) or carotenoid‑rich chromoplasts (found in yellow, orange, or red petals). ‘?’ indicates that the molecular mechanisms underlying these differentiation events in petals remain unclear.

Plastids are double-membraned organelles in plant cells. Based on their functions and pigment composition, they are classified into leucoplasts, chromoplasts, and chloroplasts [13, 14]. Leucoplasts generally lack pigments and therefore appear colorless or white. Chromoplasts primarily store carotenoids, imparting orange, yellow, or red hues to plant organs [15–17]. Chloroplasts are rich in chlorophyll, which gives plant tissues their green coloration. Chloroplast biogenesis and development in plant tissues are regulated by light, hormones, and TFs [18, 19]. Light is a key external signal regulating the differentiation of proplastids [20, 21]. In darkness, proplastids develop into etioplasts [22]. When exposed to light, etioplasts undergo extensive transformation into chloroplasts, and cotyledons and leaves turn green as protochlorophyllide (PChlide) is converted into chlorophyll (Fig. 1A) [23, 24]. Light can also induce the conversion of other plastid types into chloroplasts [25, 26]. Amyloplasts, a type of leucoplast, can convert into chloroplasts upon light exposure [27]. In potato tubers, amyloplasts in the peripheral cell layers transform into chloroplasts under illumination, leading to chlorophyll accumulation and visible greening of the tubers [28]. Light exposure induces the differentiation of chromoplasts into photosynthetically active chloroplasts in carrot roots, resulting in greening of the root [29]. After plants are exposed to light, approximately one-third of the nuclear transcriptome is altered, including many genes that encode chloroplast-targeted proteins [30, 31]. Light perception involves activation of photoreceptors, such as phytochrome A (PhyA) and PhyB, which translocate to the nucleus, regulate TF activity, and induce expression of genes related to chloroplast development and chlorophyll biosynthesis [32–35]. During this process, thylakoid membranes gradually form within plastids, promoting photosynthesis [20, 36].

Chloroplast development is regulated not only by light but also by plant hormones, such as gibberellin (GA), brassinosteroid (BR), and auxin [37–40]. Key TFs, including GOLDEN2-LIKE1 (GLK1) and GLK2, GATA nitrate-inducible carbon metabolism–involved (GNC), and cytokinin-responsive GATA factor1 (CGA1), act downstream of these signals to coordinate the process [41, 42]. Upon light exposure, these TFs are activated in etiolated seedlings, promoting the disassembly of the prolamellar body (PLB) [38, 42]. This leads to the transformation of etioplasts into chloroplasts, during which PLB membranes reorganize into thylakoids [43, 44]. The concomitant reduction of PChlide to chlorophyll and its subsequent accumulation result in leaf greening [45, 46] (Fig. 1A).

### Plastid development in green flowers

Plastid development in petals appears distinct from that in leaves. While illumination promotes the conversion of etioplasts to chloroplasts in leaves, it seems to have different impacts on the development of chloroplasts in petals at different stages. Unexposed leaf tissues contain etioplasts, which transform into chloroplasts upon illumination, initiating chlorophyll accumulation and resulting in greening of leaves. In unopened floral buds, the inner petal primordia remain enclosed by the outer sepals and thus may not be exposed to direct natural light. At this stage, chloroplasts are abundant in the petals of many species, such as peonies, peppers, and Arabidopsis thaliana, the young petals of which exhibit green coloration. In the fully opened flowers, strong sunlight may induce chloroplast damage and chlorophyll degradation. Concurrently, petal-specific developmental programs, regulated by TFs and hormones, often initiate a chloroplast-to-chromoplast transition (degreening). Chloroplasts may differentiate into leucoplasts, as observed in white petals (e.g. certain magnolia varieties), or into carotenoid-rich chromoplasts, as seen in yellow, orange, or red petals (e.g. yellow chrysanthemums, orange osmanthus, orange-red lilies) (Fig. 1B). Additionally, in certain green varieties (e.g. green carnations; Fig. 1B), petals remain green throughout development, a trait that may result from altered plastid differentiation or chlorophyll degradation pathways, whereas the exact role of light in this process remains unclear.

The process of chloroplast differentiation into chromoplasts differs between petals and leaves. This structural difference is reflected in petal chloroplasts, which typically have fewer matrix thylakoids and grana than their leaf counterparts at comparable developmental stages [47]. In some species, photosynthetically competent chloroplasts can be maintained in specific petal cell types even at maturity [48, 49]. For instance, the green spots on the Japanese gentian corolla consist of epidermal cells housing functional chloroplasts, as shown by transmission electron microscopy [50]. Similarly, carnation petals contain high levels of Rubisco large and small subunits early in development, but these subunits progressively decline during maturation, indicating a reduction in photosynthetic capacity in mature green petals [6]. Similarly, younger green petals of *Dendrobium* cv. Burana Jade exhibit much higher photosynthetic parameters than senescent petals, indicating declining photosynthetic activity with increasing flower age [51]. In the same system, leaf shading experiments reduce soluble sugar content in green petals, suggesting that these petals primarily function as carbohydrate sinks dependent on photosynthates from leaves [51]. Furthermore, green chrysanthemum petals contain photosynthetic organelles, indicating that their green color depends on undifferentiated chloroplasts and undegraded chlorophyll [5, 52].

During the transformation of chloroplasts into chromoplasts in petals, significant structural remodeling occurs, particularly in stromules and thylakoids. For example, as *Cucumis sativus* petals transition from yellow-green to dark yellow upon senescence, thylakoids disassemble and their stacked grana loosen, while the number of tubular stromules increases [53]. Similarly, the yellow-green petals of *Tropaeolum majus* L. contain chloroplasts that are actively differentiating, characterized by few, irregular grana [54]. Throughout petal opening, the thylakoid membranes within these plastids are gradually degraded [54].

The differentiation of chloroplasts into chromoplasts coincides with chlorophyll degradation, driving a shift in petal pigmentation [55]. For instance, during this transition in *Lilium longiflorum* flower buds, carotenoid content increases fivefold while chlorophyll content drops to one-fifth, causing petals to turn from green to yellow [55]. Similarly, the green-to-white transition in Arabidopsis petals is precisely regulated by hierarchical networks and influenced by developmental and environmental cues [1]. Early flower buds initially appear green due to abundant chloroplasts; upon flower opening, the distal parts of petal cells rapidly expand, chloroplasts transition to non-photosynthetic plastids, and chlorophyll is swiftly degraded [1], while the proximal region retains some chloroplasts and therefore remains partially green [56]. In *Gentiana lutea*, the young flower buds exhibit a dark green coloration, accompanied by active chloroplast biogenesis. As petal development proceeds, chloroplasts gradually differentiate into chromoplasts, with a concomitant increase in carotenoid accumulation, leading to a transition in petal color to golden yellow [57]. The downregulation of the plastid division gene *Filamentous temperature-sensitive Z* (*FtsZ*) during this process provides molecular evidence for the structural remodeling from chloroplasts to non-photosynthetic plastids [57]. Additionally, petals of peppers, lilies, *Kalanchoe blossfeldiana*, and *Orychophragmus violaceus* are initially green, containing chloroplasts. During the development of flowers, chloroplasts are differentiated into various types of plastids. This transformation is followed by the accumulation of other pigments within petal cells, such as carotenoids or anthocyanins, resulting in diverse floral colors [1].

Collectively, these above findings indicate that petal chloroplasts follow an organ-specific developmental program distinct from that in leaves. The core cytological basis for petal color variation lies in their programmed transformation into other plastid types (e.g. chromoplasts) [54, 55]. This transformation is a dynamic and tightly regulated process, shaped by developmental stage, light exposure, and tissue-specific programs. It is systematically accompanied by chlorophyll degradation and the accumulation of other pigments (e.g. carotenoids). The final floral color depends on the precise balance and overlay of these events, where green color depends on chlorophyll and chloroplasts, while other colors are determined by the specific pigments and plastids.

## Chlorophyll metabolism and green flower formation

The precise regulation of chlorophyll biosynthesis and degradation is fundamental for plant organs to fulfill their specialized functions. A tightly regulated balance between these two processes is crucial for healthy plant growth and environmental adaptation. Mutation of a key gene encoding a chlorophyll metabolic enzyme disrupts this balance, leading to abnormal chlorophyll accumulation. This imbalance manifests as either chlorophyll deficiency or retention, causing plant organs to appear pale-yellow or remain persistently green.

### An overview of the mechanism of chlorophyll metabolism

Although chlorophyll biosynthesis has been extensively studied, research remains largely centered on leaf tissues. The processes of chlorophyll biosynthesis, cycling, and degradation collectively determine chlorophyll content. Chlorophyll is synthesized via the branched tetrapyrrole pathway. This pathway begins with glutamyl-tRNA and proceeds through 15 enzymatic reactions to produce Chl a, involving more than 10 enzymes encoded by over 20 genes [58, 59]. The biosynthesis steps are briefly described as follows. The first biosynthetic stage comprises the conversion of glutamyl-tRNA to protoporphyrin IX (Proto IX) via nine enzymatic steps. These steps start with the reaction of glutamyl-tRNA synthetase (GluRS), glutamyl-tRNA reductase (GluTR), and glutamate-1-semialdehyde 2,1-aminomutase (GSAAT) to synthesize 5-aminolevulinic acid (ALA). Subsequently, 5-aminolevulinate dehydratase (ALAD), hydroxymethylbilane synthase (HMBS), and uroporphyrinogen III synthase (UROS) catalyze the formation of uroporphyrinogen III. This is followed by the actions of uroporphyrinogen III decarboxylase (UROD), coproporphyrinogen III oxidase (CPO), and Proto IX oxidase (PPO) to produce Proto IX. The second biosynthetic stage is the conversion of proto IX into chlorophyll. First, magnesium chelatase (MgCh) inserts Mg^2+^ into proto IX to form Mg-proto IX [60, 61]. Mg-proto IX methyltransferase (CHLM) catalyzes Mg-proto IX to form Mg-proto IX monomethylester. Subsequently, Mg-proto IX monomethylester is converted to PChlide via Mg-PIX monomethylester cyclase (CHL27). The subsequent formation of chlorophyllide a (Chlide a) involves two key reductions, the central, light-dependent reduction of PChlide catalyzed by PChlide oxidoreductase (POR) [62], and the reduction of the 8-vinyl group by divinyl reductase (DVR). Chlorophyll synthase (CHLG) converts Chlide a into Chl a. Subsequently, Chl a can be reversibly converted back to Chlide a by Chl dephytylase 1 (CLD1). Furthermore, Chl a is converted to Chl b by Chlide a oxygenase (CAO) [59, 63, 64]. Chl b can revert to Chl a via Chl b reductase (CBR) and 7-hydroxymethyl Chl a reductase (HCAR). This cyclic conversion between Chl a and Chl b is known as the ‘chlorophyll cycle’ [65].

In addition to dynamic cycling, chlorophyll degradation plays a critical role during tissue aging. Chl b is initially reduced to Chl a through the ‘chlorophyll cycle’. The subsequent degradation of Chl a to pheophorbide a (Pheide a) is known to proceed via two potential pathways. In one pathway, dephytylation catalyzed by chlorophyllase (CLH) occurs prior to the removal of the central Mg^2+^ [66]. Alternatively, Mg^2+^ extraction may precede dephytylation, leading to the formation of pheophytin a (Phein a) as an intermediate [67]. In this pathway, the first committed step is the irreversible removal of Mg^2+^ from Chl a [68]. This reaction is catalyzed by magnesium-dechelatase, encoded by the *Stay-Green*/*Non-Yellowing* (*SGR*/*NYE*) [69]. Notably, dysfunction in senescence-associated genes such as *CBR*, *HCAR*, or *SGR* leads to a stay-green phenotype [70–73]. Subsequently, Phein a undergoes dephytylation catalyzed by the enzyme pheophytinase (PPH), yielding Pheide a. Next, Pheide a is converted through pheophorbide a oxygenase (PAO) and RCC reductase (RCCR) into the primary fluorescent Chl catabolites (*p*FCCs) [59]. Modified *p*FCCs are transported into vacuoles and converted to nonfluorescent Chl catabolites (NCCs) [74].

### Genes of chlorophyll metabolism pathways influence green flower formation

Chlorophyll is the primary pigment responsible for green coloration in flowers, as seen in green varieties of *Cymbidium* orchid, carnation cultivars and snowdrop (*Galanthus nivalis* L*.*) [75–77]. Its accumulation and metabolism in petals are crucial for green flower formation. Current research indicates that the green petal phenotype is maintained by a dynamic equilibrium between chlorophyll biosynthesis and degradation. Disruption of this balance triggers rapid chloroplast remodeling into leucoplasts or chromoplasts, accompanied by the deposition of carotenoids, anthocyanins, or other pigments in petal cells, thereby altering flower color.

**Figure 2:**
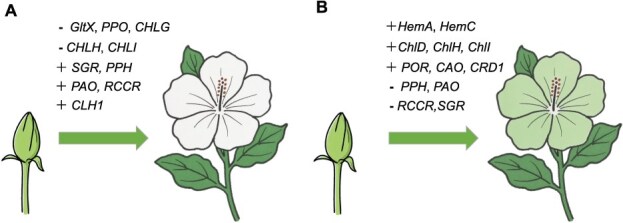
The green petal phenotype is maintained by a dynamic equilibrium between chlorophyll biosynthesis and degradation pathways. (A) Reduced biosynthesis combined with elevated degradation leads to chlorophyll loss and petal degreening [7, 9, 12, 78]. (B) Enhanced biosynthesis together with suppressed degradation promotes chlorophyll accumulation and maintains green pigmentation [7, 8, 79, 80]. (`+' and `-' indicate higher and lower transcript abundance, respectively).

Substantial chlorophyll loss in petals correlates with the downregulation of chlorophyll biosynthetic genes and/or the upregulation of degradation-related genes (Fig. 2A). Consequently, the floral color shifts away from green with the simultaneous accumulation of other pigments. The shift in flower color of *Lonicera japonica* Thunb., particularly the transition from green to white, is driven by chlorophyll depletion. Comparative transcriptome analysis demonstrated that this progressive loss of green pigmentation is linked to concurrent downregulation of chlorophyll biosynthesis genes (*GltX*, *PPO*, *CHLG*) and upregulation of degradation genes, like *PPH*, *PAO,* and *RCCR* [7]. Similarly, in carnation (*Dianthus caryophyllus* L.), microarray and RT-qPCR analyses revealed that compared with green petals, non-green petals exhibit chlorophyll deficiency, which correlates with lower expression of chlorophyll biosynthesis genes (e.g. *CHLH*/*CHLI*) and higher expression of degradation genes (such as *SGR* and *PPH*) [78]. In cabbage and rapeseed, petals accumulated less chlorophyll and had a lower Chl a/b ratio compared to leaves. qRT-PCR analysis indicated that this was correlated with significantly lower expression of chlorophyll biosynthesis genes in immature petals, especially those involved in the late steps of the pathway (*CHLD* to *CAO*) [81]. Beyond mere correlation, the dynamic process and functional importance of chlorophyll degradation are demonstrated in the *Paeonia suffruticosa* ‘Lv Mu Yin Yu’ cultivar, which exhibits a striking bright-green corolla at early bud opening that rapidly fades to pale pink at full bloom [12]. Functional evidence come from VIGS experiments showing that silencing of *PsCLH1* yields greener petals with elevated chlorophyll content, demonstrating that *PsCLH1* promotes chlorophyll degradation and petal whitening [12]. Notably, the role of the chlorophyll degradation gene *SGR* appears crucial and conserved across species. Supporting this, transcriptomic analysis in *Edgeworthia chrysantha* demonstrated that its sequential flower color transition—from green to yellow to white—is closely associated with *SGR* expression [9].

The tulip tree (*Liriodendron tulipifera*) is horticulturally prized for its flowers, which feature distinct orange bands at the petal bases. Transcriptomic analysis demonstrated that this pattern is driven by localized chlorophyll degradation, and is associated with the precise modulation of key carotenoid genes including carotenoid isomerase (*CRTISO*) and ε-lycopene cyclase (*ε*-*LCY*), together with the downregulation of chlorophyll biosynthetic genes [82]. Similarly, the striking color patterning of petals is tightly correlated with fine-tuned modulation of local gene expression. For instance, in the Asiatic hybrid lily cultivar ‘Padhye’, petals transit from green to white at the tip and from green to purple at the base during development. Spatiotemporal transcriptome analysis revealed that the white upper tepals exclusively exhibited high expression of four chlorophyll degradation genes (*PPH*, *PAO*, *RCCR*, and *SGR*). The purple basal tepals showed high expression of these four genes, additionally accompanied by high expression of seven genes in the anthocyanin biosynthetic pathway (e.g. *CHS*, *F3′H*, and *DFR*) [80]. The expression patterns of *LhMYB12-Lat* and *LhWRKY44* were similar to those of anthocyanin biosynthetic genes (e.g. *DFR* and *CHS*). However, their role in lilies remains unelucidated and thus requires further study [80].

Petal greening with higher chlorophyll content is associated with the coordinated upregulation of chlorophyll biosynthetic genes and downregulation of chlorophyll catabolic genes (Fig. 2B). RNA-seq analyses indicated that green petal formation in the carnation cultivar ‘Seychelles’ was correlated with the upregulation of chlorophyll biosynthetic genes (e.g. *CHL*, *CHLM*, and *PORA*) and elevated chlorophyll accumulation [83]. Transcriptomic analysis of *Prunus serrulata* petals demonstrated a higher chlorophyll content in this tissue, correlating with the upregulated expression of core chlorophyll biosynthesis genes (e.g. *HemA*, *HemC*, *ChlD*, *ChlH*, *ChlI*, *POR*, and *CAO*) [8]. Based on transcriptomic data, the accumulation of pigment in green sepals of *Hydrangea macrophylla* ‘green with blue edges’ strongly correlates with the upregulation of chlorophyll biosynthesis genes—particularly the marked elevation in the transcriptional levels of *PORA* and *CRD1* [79]. Conversely, in *L. japonica* petals, green pigmentation during early development correlates with the downregulation of core catabolic genes (*PAO*, *PPH*, *RCCR*) [7]. The early phase of color persistence, correlated with low catabolic gene expression, represents a critical, shared stage in floral development, even in organs destined to display complex patterns. For example, in the bicolor tepals of *Lilium* ‘Tiny Padhye’, the early green stage correlated with reduced transcript levels of major degradation genes (*PPH*, *PAO*, *RCCR*, *SGR*), especially in the lower tepal parts [80]. Across these examples, correlative evidence links chlorophyll-related gene expression to green pigmentation.

It is worth mentioning that the pigmentation of the petal band in *L. tulipifera* is a finely tuned two-step regulatory process. First, prior to pigment accumulation, the band area is pre-defined through local repression of chlorophyll biosynthesis (`degreening'). Subsequently, the carotenoid biosynthesis pathway is specifically activated in this region, leading to the accumulation of orange pigments. This spatiotemporally separated regulatory model provides deeper insights into the formation of complex patterns in plant floral organs [82]. The study delivers the first-ever integrated profiling of metabolomic and transcriptomic changes during floral pigmentation in the tulip tree, offering valuable targets for breeding and metabolic engineering while advancing floral evolution research [82]. Collectively, these studies demonstrate that green petal coloration arises from a finely tuned balance between chlorophyll biosynthesis and degradation, where even subtle shifts determine the outcome toward either chlorophyll retention or loss. In most non-green petals, this balance is skewed toward rapid degradation and reduced synthesis, whereas green-flowered species suppress catabolism and/or enhance biosynthesis to prolong chlorophyll presence. The diversity of expression patterns across species and even within different petal regions underscores that multiple regulatory strategies can converge on a similar visual phenotype.

## Regulation of chlorophyll metabolism by TFs

TFs regulate chlorophyll biosynthesis and degradation pathways, thereby influencing chlorophyll content in plants. They act by binding to specific DNA sequences in the promoters of chlorophyll-related genes and modulating their expression in response to developmental and environmental cues.

### An overview of TFs regulating chlorophyll metabolism

Extensive studies have demonstrated that chlorophyll metabolism is regulated by environmental factors and plant hormones. Various environmental cues influence chlorophyll content in angiosperms by modulating chlorophyll metabolic pathways [84]. TFs respond to these cues by altering their own expression or activity, which in turn modulates the expression of functional genes involved in chloroplast development and chlorophyll metabolism [20]. Thus, via this TF-mediated regulatory cascade, plants fine-tune chlorophyll biosynthesis and photosynthetic capacity to acclimate to diverse environmental conditions [20].

Key TFs such as GLK1/2, PIFs, EIN3, and ABI5 play a central role in regulating chlorophyll biosynthesis and degradation [85, 86]. Light-mediated TFs can either activate or repress chlorophyll biosynthesis. They respond to light signals and regulate target genes by binding to promoter regions involved in the chlorophyll biosynthetic pathway. PIFs are bHLH-family TFs that directly interact with phytochromes. They play central roles in photomorphogenesis [87–89]. In darkness, negative regulators like PIFs and COP1 accumulate. PIFs directly suppress tetrapyrrole synthesis genes, while COP1 degrades elongated hypocotyl 5 (HY5) [90–93]. Both actions inhibit chlorophyll biosynthesis and chloroplast development [90–93]. Upon illumination, photoreceptors phyA/B, cryptochrome 1/2 (CRY1/2) trigger the degradation of PIFs and inactivate COP1, allowing positive regulators such as HY5 and Far-red elongated hypocotyls 3 (FHY3)/Far-red impaired response (FAR1) to accumulate [41, 94]. These regulators activate GLK1/2 and downstream genes to promote chlorophyll biosynthesis and chloroplast maturation, enabling the transition from etiolation to greening [41].

Hormonal signaling also integrates with light signals to control chlorophyll biosynthesis, often through shared core TFs, such as GLK1/GLK2 and CGA1 [95]. This deep integration allows the plant's internal physiological state, dictated by its hormone balance, to constantly modulate its response to external environmental cues, thereby enabling adaptive and context-dependent control of chlorophyll accumulation. Upon first exposure to light, ethylene promotes chlorophyll biosynthesis in the cotyledons of young seedlings [96]. During the transition from skotomorphogenesis to photomorphogenesis, the EIN3/EIL1 and PIF1 cooperatively induce the expression of *POR* genes to promote seedling greening and protect cotyledons from photobleaching in Arabidopsis [95, 96]. Similarly, DELLA proteins, negative regulators of GA signaling, alleviate PIF repression under dark conditions. This directly induces *POR* expression and protein accumulation, thereby maintaining elevated POR enzymatic activity [97]. Additionally, auxin-responsive factors (e.g. ARFs) and cytokinin-responsive B-type ARRs bind to promoters of genes like *GLK1* and *Chl a/b-binding protein* (*CAB*), promoting chlorophyll biosynthesis [18, 98, 99].

### TFs regulate chlorophyll accumulation in green flowers

**Figure 3:**
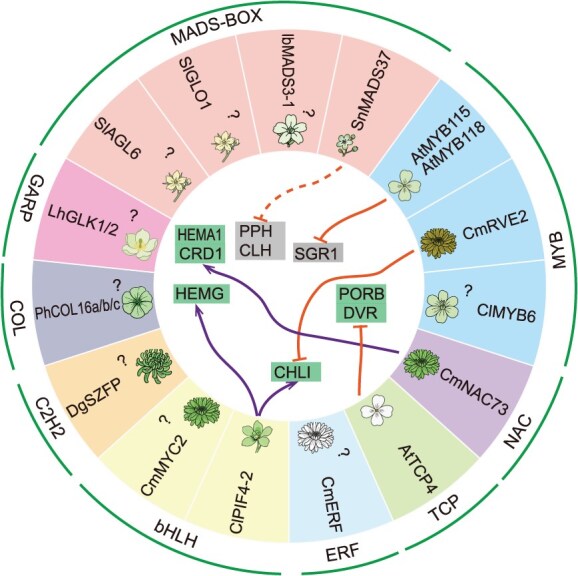
Transcriptional regulation of chlorophyll metabolism and development in floral organs. TFs orchestrate chlorophyll accumulation in floral organs by modulating transcriptional networks controlling chlorophyll metabolism, chloroplast biogenesis, and floral organ development. Arrows: TFs upregulate chlorophyll biosynthesis genes, including *HEMG*, *CHLI*, *HEMA1*, and *CRD1* [100, 102]. T-bars: TFs suppress *PORB*, *DVR*, *CHLI*, and *SGR* in the chlorophyll metabolic pathway [1, 101, 104]. Dashed T-bars: Putative suppression of *PPH* and *CLH* by TFs [105]. ‘?’: unverified regulatory targets.

In the green petals of certain plant species, TFs play a central role in regulating chlorophyll accumulation. For instance, the green coloration of *Cymbidium lowianum* sepals and petals at the bud stage is attributed to PIF4–2-mediated upregulation of chlorophyll biosynthesis genes (*HEMG* and *CHLI*), as supported by evidences from dual-luciferase and yeast one-hybrid assays [100]. In chrysanthemum ‘Lv Dingdang’, high-intensity light induced loss of green coloration during flower opening, while low-intensity light maintained it. The MYB transcription factor Reveille2 (CmRVE2) acts as a signaling node that integrates environmental and hormonal cues to regulate green petal formation [101]. Its expression is suppressed under low light or by GA but induced by highlight and ABA. Upon upregulation, CmRVE2 inhibits chlorophyll biosynthesis in petals by directly binding to the promoter of *CHLI1* and repress its expression [101]. Beyond this light-hormone integrated signaling module, chlorophyll accumulation in chrysanthemum florets is also subjected to direct transcriptional control by specific factors. Comparative transcriptome analysis revealed that chlorophyll accumulation in the outer whorls of chrysanthemum ray florets was associated with the TF CmNAC73. Functional studies confirmed its positive role: transient overexpression of *CmNAC73* enhanced chlorophyll biosynthesis, and combined transactivation and yeast one-hybrid assays demonstrated its direct binding to the promoters of *HEMA1* and *CRD1* [102]. In addition to the mechanistic evidence for CmNAC73, correlative studies point to the involvement of other TFs. In several chrysanthemum cultivars, including *Chrysanthemum vestitum*, *Chrysanthemum morifolium* ‘Chunxiao,’ and ‘Green Anna’, chlorophyll accumulation in petals showed a positive correlation with the expression level of *CmMYC2*, a bHLH family TF. This correlation suggests that CmMYC2 likely functions to enhance chlorophyll accumulation in floral organs [103]. The above findings are summarized in Fig. 3.

In chrysanthemum, petal chlorophyll homeostasis is determined not by individual regulators but by the concerted action of TFs from multiple families. This regulatory complexity is evidenced by RNA sequencing and qRT-PCR analyses, which revealed that elevated chlorophyll content in green-flowered varieties (compared to white ones) is associated with higher expression of key regulatory genes such as *CmCOLa*, *CmCOLb*, *CmERF*, and *CmbHLH* [52]. Beyond these specific positive regulators, chlorophyll levels are governed by a dynamic equilibrium between biosynthesis and degradation. Comparative transcriptome analysis of white and green chrysanthemum petals revealed that chlorophyll deficiency in white petals was associated with the coordinated downregulation of biosynthetic genes and upregulation of catabolic genes. Furthermore, co-expression network analysis revealed that chlorophyll levels correlated positively with the expression of *ERF* and *Constans16-like* (*COL16-like*) [5].

Studies in other species confirm that additional TF families, including MYB and TCP, play key roles in determining petal chlorophyll levels. For example, mutation of the R2R3-MYB TF gene *MYB6* (*ClPC*) in watermelon shifts petal color from yellow to yellow-green [106]. Transmission electron microscopy revealed that mutant petals contained numerous chloroplasts with fully developed stroma and thylakoid membranes, structures that were absent in the wild-type petals. Consistent with this, transcriptomic data showed elevated expression of chlorophyll biosynthesis genes (e.g. *CHLM* and *POR*) in the mutant [106]. TCP TFs act as important negative regulators of chlorophyll biosynthesis and petal greening in Arabidopsis. In the *tcp* multi-mutant, key biosynthesis genes (*PORB* and *DVR*) and the positive regulator *SOC1* are derepressed. This derepression promotes chlorophyll production and leads to the formation of green petals [1].

Genetic engineering approaches have been used to modulate petal chlorophyll metabolism by manipulating specific TFs, yielding transgenic flowers with pale green coloration [107]. For instance, the COL TF family regulates photoperiod response, flowering time, and chlorophyll metabolism [108]. In petunia, corolla chlorophyll content positively correlated with the transcript levels of three *COL16* homologs (*PhCOL16a*, *PhCOL16b*, *PhCOL16c*) [107]. Overexpression of *PhCOL16a* significantly elevated chlorophyll levels and upregulated key biosynthetic genes (e.g. *HEMA*, *CHLH*, *CHLM*, *CRD*, *PORC*, *CHLG*, *CAO*), resulting in light-green corollas [107]. In another example, expressing *AtMYB115* and *AtMYB118* under floral-organ-specific promoters in Arabidopsis suppressed the chlorophyll degradation gene *SGR1*, leading to pale green petals and causing stamen sterility (Fig. 3) [104]. Furthermore, GLK TFs are master regulators of chloroplast development. Overexpression of *Liriodendron Hybrid LhGLK1* in Arabidopsis elevated chlorophyll levels in rosette leaves and induced the formation of ectopic chloroplasts in primary roots and petal vasculature, resulting in aberrant greening of these typically non-photosynthetic tissues [109].

In the aforementioned study, the role of TCP as a ‘greenness suppressor’ has been clearly established. Furthermore, the spatiotemporal expression pattern of TCP4, specifically localized in the distal region of petals during flower developmental stages 9–12, closely coincides with the occurrence of distal petal ‘degreening’ [1]. Overall, the above studies highlight that TFs act as pivotal nodes linking environmental and hormonal cues to chlorophyll metabolic pathways, with their coordinated activity determining whether petals undergo rapid degreening or sustain chlorophyll accumulation to maintain a green phenotype. Furthermore, these findings provide promising targets for manipulating floral greening.

## Homeotic transformation from petals to sepals: the green flower phenomenon under gene regulation

The ABCDE genes are fundamental to floral organ identity and development. Mutations in these genes can disrupt normal developmental pathways, leading to abnormal morphologies. Beyond organ identity, the formation of ‘calyx-shaped green flowers’ is driven by specific expansin genes, which facilitate sepal enlargement and their transition into petaloid structures.

### B-class gene defects disrupt floral organ identity

**Figure 4:**
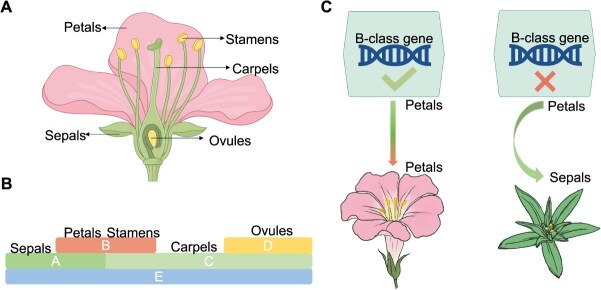
Molecular basis of floral organ development and a related phenotype. (A) Diagram depicting floral morphology. (B) Illustration of the classical ABCDE model for floral organ identity specification. (C) Model of the homeotic conversion caused by B-class gene loss, where petals transform into sepals and produce a ‘green flower’ phenotype.

Floral organ identity and morphogenesis are orchestrated by the class A-E genes, most of which encode MADS-box TFs (Fig. 4A and B). Their function is mediated by specific protein complexes. Consequently, any dysfunction in these genes can result in abnormal floral organ development.

The class B genes *APETALA3* (*AP3*) and *PISTILLATA* (*PI*) are key determinants of petal and stamen identity in Arabidopsis, functioning through their expression in the second and third floral whorls. Mutations in these genes disrupt organ identity, leading to homeotic conversions [11, 110, 111]. In Petunia, the homologs *DEFICIENS* (*DEF*) and *GLOBOSA* (*GLO*) function analogously to AP3 and PI, respectively [112, 113]. Their encoded proteins form heterodimers to jointly regulate petal and stamen development. Mutations in *PhDEF* result in petals being replaced by sepals, forming unique flowers with all-green sepals [112, 113] (Fig. 4C). In tomato, silencing of *SlGLO1* leads to green, reduced-size petals and malformed stamens. This phenotype coincides with upregulated expression of chlorophyll biosynthesis genes and elevated chlorophyll levels, indicating that *SlGLO1* represses petal degreening [114]. Similarly, the petunia green petal (*gp*) mutant (line PLV) exhibits a homeotic transformation of petals into sepals [115]. The petal defect in this *gp* null mutant can be rescued by expressing *pMADS1* from the CaMV 35S promoter, confirming *pMADS1*’s essential role in petal formation [115]. Likewise, mutation in the *California Poppy GLO*/*PI* homolog (*sei*-*1*) causes the transformation of petals into sepals and stamens into carpels, displaying a classic class B mutant phenotype [116]. Furthermore, silencing the *Ranunculaceae AP3-3* gene also produces green, sepaloid petals [117].

B-class MADS-box genes (or their dimeric protein complexes) can influence chlorophyll content in floral organs such as petals through two pathways. (i) Chromatin immunoprecipitation revealed that PI protein directly binds and represses the promoters of *GNC* and *GNL*, thereby attenuating chlorophyll biosynthetic capacity [118]. (ii) Negative regulation of the *BANQUO* family (*BNQ1*/*BNQ2*/*BNQ3*), which are required for chlorophyll biosynthesis. In Arabidopsis, AP3 and PI ensure the proper differentiation of petals by specifically downregulating the expression of the *BNQ*, thereby inhibiting chlorophyll accumulation in these floral organs [119]. Collectively, defects in B-class genes lead to abnormal petal greening primarily through two interconnected mechanisms: by inducing homeotic transformations (petal-to-sepal) and by directly derepressing chlorophyll biosynthetic pathways.

### E-class genes as key regulators of petal identity and chlorophyll metabolism

E-class genes are essential for the function of B-class and C-class genes. In the *Sepallata1*/*2*/*3*/*4* (*sep1*/*2*/*3*/*4*) quadruple mutant Arabidopsis, all floral organs are replaced by green spiral leaf-like structures [120]. In *Solanum nigrum*, the *SEP3*-*like* gene *SnMADS37* has been identified as a pivotal regulator of petal development [105]. Its overexpression disrupts floral organ development and promotes chlorophyll accumulation. In these lines, pronounced chlorophyll retention, particularly at the distal ends of petals (Fig. 3), resulted from the downregulation of *SnCLH* and *SnPPH* expression [105]. Similarly, *AGL6* and its homologs function like *SEP* genes. Silencing *SlAGL6* in tomato leads to fused sepals, green petals, and reduced petal size [121]. This is accompanied by the upregulation of *SlGLK1*/*2* and their downstream targets (*SlrbcS3B*, *SlCab-7*), increased chlorophyll levels, and altered petal epidermal cell morphology, collectively indicating that *SlAGL6* coordinates sepal–petal identity specification and chlorophyll homeostasis [121]. Furthermore, partially inhibited *FBP2* gene expression caused the morphogenesis of calyx-like tissue in the corolla. Consistent with this green phenotype, cDNA microarray analysis indicated that expression levels of several photosynthesis-related genes—including the gene encoding the small subunit of Rubisco—were significantly higher in these tissues compared to wild-type purple petals [122]. Overall, dysfunction or misregulation of E-class genes contributes to abnormal floral greening by disrupting organ identity (often causing leaf-like transformations) and by directly or indirectly dysregulating chlorophyll metabolic genes.

### Abnormal sepal growth leading to ‘calyx-shaped green flowers’

**Figure 5:**
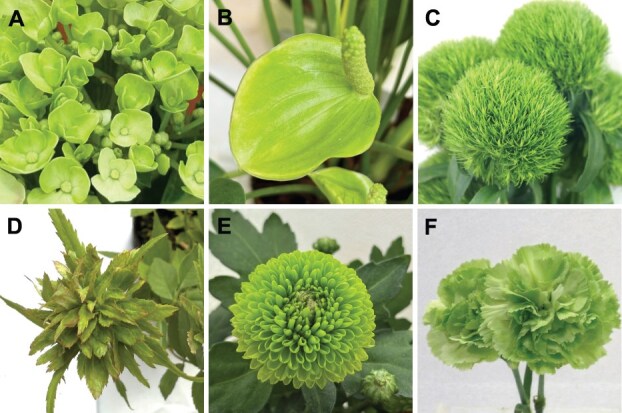
Special green-flowered cultivars. (A) *Hydrangea* ‘Mini Green’ (B) *Anthurium andraeanum* Linden (C) *Dianthus* ‘Green Trick’ (D) *Rosa chinensis* cv*.* ‘Viridiflora’ (E) *Chrysanthemum* ‘Green Pingpong’ (F) *Dianthus caryophyllus* ‘Mint Green’.

Excessive expansion of sepals can also lead to the formation of ‘calyx-shaped green flowers’ [123]. A notable example is found in *H. macrophylla*, where the showy ornamental structures are not true flowers but enlarged, pigmented, petal-like sepals (Fig. 5A). Integrated genomic and transcriptomic analyses have revealed that the morphological conversion of sepals into petaloid structures is driven by specific members of the expansin gene family, notably *HmEXPA4*, *HmEXPA15*, and *HmEXPA17*. These expansins promote sepal enlargement by facilitating cell wall loosening, ultimately contributing to the formation of these petal-like organs [123]. Furthermore, *Anthurium andraeanum* Linden, *Dianthus* ‘Green Trick’ and *Rosa chinensis* cv*.* ‘Viridiflora’ exhibit striking sepaloid green flowers due to abnormal sepal growth (Fig. 5B–D). The mechanisms underlying these aberrant floral organs are not very clear.

As mentioned above, deficiencies in both B-class and E-class genes can lead to green flowers by inducing sepaloid petals and disrupting chlorophyll repression. In contrast, while B-class mutations are largely confined to petals and stamens, loss of E-class function can cause a complete reversion of the entire flower to a green, leaf-like state. Therefore, the future implementation of a ‘Green Flower Project’ would require ensuring that introduced color-modulating genes do not interfere with the function of these critical organ-identity genes [112, 113]. Collectively, the developmental reprogramming that converts petals into sepaloid or leaf-like structures often concurrently preserves or enhances chlorophyll content, thereby producing green floral organs. These findings highlight a close developmental coupling between floral organ identity and pigment regulation, indicating that perturbations in one pathway can simultaneously alter both morphology and coloration.

## Other factors affecting green flower formation

### Pleiotropic effects and sepaloid transformations induced by *SUP* and *STMADS* genes

*Superman* (*SUP*) genes play important roles in flower development, plant growth, and morphogenesis [124]. The chrysanthemum gene *DgSZFP* is a homolog of *SUP*. Its overexpression in tobacco elicited pleiotropic developmental alterations, including significantly increased petal tube diameter, seed size/weight, and chlorophyll concentration compared to the wild type. These findings indicate that *DgSZFP* promotes chlorophyll accumulation and regulates floral organ morphology, likely in a synergistic manner with other factors [125]. Similarly, ectopic expression of *IbMADS3-1* (a member of the *STMADS* subfamily from *Ipomoea batatas*) in tobacco led to chlorophyll accumulation and a sepaloid transformation in the petals of transgenic plants [126].

### Roles of *SOC1*-*like* genes and chlorophyll-associated proteins in green petal development

Green flower formation is controlled not only by organ identity genes but also by flowering signal integrators that regulate chlorophyll metabolism. For instance, *SOC1-like* genes display pleiotropic effects: they modify petal cell morphology while promoting chloroplast biogenesis under environmental stress. In addition, the light-harvesting Chl a/b binding (Lhc) proteins in plastids were shown to be important for maintaining green pigmentation.

*SOC1*/*SOC1*-*like* genes commonly function as ‘flowering integrators’ across plant species [127]. In *Gerbera hybrida*, ectopic overexpression of *GhSOC1* resulted in shorter ray florets with irregular epidermal cells. These transgenic petals also displayed a greenish abaxial surface and deeper adaxial coloration compared to the wild type [128]. Furthermore, the green color in petals of transgenic tobacco and Arabidopsis plants is induced by the combined effect of heat stress and the overexpression of *SOC1* or its homologs (e.g. *FPB21* and *FBP22*) [129]. Transgenic and transcriptomic analyses suggest that in petals, heat stress may induce GATA TFs and upregulate chlorophyll biosynthetic genes (*CHL27*, *POR*, and *CAO*), which collectively promote chloroplast biogenesis and chlorophyll accumulation [129]. Consistent with findings in tobacco, transgenic petunia plants overexpressing *FPB21* similarly developed light green petals under heat stress conditions [130]. Although transcriptomic evidence confirms the role of *SOC1*/*SOC1-like* genes in promoting chloroplast biogenesis, the mechanisms by which SOC1 proteins exert this control are unknown. Identifying the direct targets and downstream partners of SOC1 in this context is therefore an important next step. Beyond transcriptional regulation, the light-harvesting complex is an important contributor to green petal formation. For example, functional studies of *PsLhcb1* and *PsLhcb5* with the overexpression and gene silencing in transgenic tobacco and the petals of *P. suffruticosa* ‘Lv Mu Yin Yu’ showed increase of chlorophyll content or petal degreening, respectively [12].

### Regulation of green flower formation by phytohormones

Phytohormones also modulate petal chlorophyll accumulation. In *L. japonica*, the petal color transition is accompanied by marked declines of indole-3-acetic acid (IAA), zeatin riboside (ZR), GA3, BR, and methyl jasmonate (MeJA), while ABA rises significantly [7]. This hormonal shift suggests that these compounds are important regulators initiating petal degreening. Supporting this role, exogenous application of MeJA can delay the opening of tree peony cut flowers [131] and improve the postharvest value of *Eustoma* [132]. Double-flowered *Eustoma* frequently exhibit irregular pigmentation at the bud stage, with persistent chlorotic patches at the petal tips. Exogenous MeJA application markedly diminished these green areas. Even brief postharvest MeJA exposure mitigated color unevenness in early-harvested ‘Voyage (Type II) Blue’ flower buds, yielding consistent coloration [132]. Therefore, MeJA emerges as a versatile plant growth regulator in floriculture. By concurrently modulating flower opening kinetics and promoting uniform petal coloration, it serves as a valuable tool for enhancing the postharvest quality and marketability of cut flowers. Further research could optimize application protocols (concentration, timing, and method) for different species and cultivars.

### PH affects the formation of green flowers

Although pH does not directly alter chlorophyll color, it indirectly affects its visual prominence by influencing other pigments. Under neutral or weakly alkaline conditions, flavonoids (e.g. flavonols) appear yellow or colorless, thereby enhancing the visibility of green. Conversely, under acidic conditions, they may turn red or purple, overlapping with and masking the green hue. In snapdragon (*Antirrhinum majus* ‘Legend White’) buds, chloroplast-rich petals appear green initially [133]. During the plastid-to-chromoplast transformation, autophagy mediated the rapid degradation of starch, chlorophyll, and carotenoids, causing the green color to fade. Subsequent intracellular pH shifts and phenolic oxidation then established the primary color palette, transitioning petals from green to pale yellow gradients [133]. Another example is found in *Puya alpestris* (*Bromeliaceae*), whose flowers exhibit a unique color gradient shifting from green-blue at the tip to pure blue at the base. This distinctive coloration arises from three key factors: (i) intermolecular copigmentation between delphinidin 3,3*′*,5*′*-tri-O-glucoside and accessory pigments (e.g. luteolin 4*′*-O-glucoside) that produces a stable blue hue; (ii) the yellow color contributed by myricetin 3,3*′*,5*′*-tri-O-glucoside under relatively high pH conditions; and (iii) the underlying green contribution from chlorophyll [134].

As mentioned above, there is an association between the dynamic changes in endogenous hormones and petal degreening. In the future, exogenous hormone treatments (e.g. MeJA) could be a promising postharvest technique to improve the commercial quality of cut flowers, especially for cultivars prone to irregular or abnormal pigmentation [132].

## Prospects

Green flowers offer more than ornamental novelty, and they are valuable for elucidating the regulation of petal pigment metabolism and gene expression. Thus, they constitute a key model system for investigating how chlorophyll biosynthesis, plastid development, and transcriptional networks are integrated within the specialized developmental context of floral organs, driving discoveries in plant biology and breeding.

### Multifunctional adaptation strategies and advantages of green flowers

Why do green flowers persist despite their low chromatic contrast with foliage—a trait theoretically disadvantageous for pollinator attraction? Although relatively rare, they nonetheless exist [135]. Compared to pure green flowers, those with yellow-green hues present greater chromatic contrast, potentially improving visibility [136]. Research suggests that in green flowers, the accumulation of carotenoids as accessory pigments may enhance their visual salience to specific pollinators [136]. The green coloration itself may also confer advantages, such as reducing herbivore pressure and maintaining photosynthetic capacity [6, 51, 135]. Understanding these multifunctional traits is essential for a mechanistic perspective on floral adaptation, as it reveals how multi-trait selection, driven by non-pollinator agents, shapes floral diversity and advances our understanding in evolutionary ecology. To fully understand these trade-offs, we will need to quantitatively analyze green-flowered species using integrated, multidisciplinary approaches. This entails evaluating the fitness contributions of photosynthesis, pigment metabolism, and herbivore avoidance against the potential costs in pollinator attraction.

### Developmental and hormonal regulation of chlorophyll retention

What are the molecular mechanisms by which phytohormonal networks orchestrate intrinsic developmental programs to control chlorophyll catabolism and plastid type conversion in petal cells? In most flowering plants, the maturation of floral buds follows a default developmental pathway whereby chloroplasts within petal cells convert into chromoplasts or leucoplasts [1, 56]. This process is modulated by intrinsic developmental and hormonal signals. Phytohormones such as ethylene, abscisic acid (ABA), and jasmonate (JA) are known to promote chlorophyll degradation [137–139]. In contrast, cytokinin accelerates the greening process. It promotes the structural conversion of etioplasts to chloroplasts, accompanied by characteristic ultrastructural changes, and concurrently elevates the steady-state levels of tetrapyrrole biosynthesis intermediates, thereby enhancing chlorophyll production [18].

In *Prunus serrulata*, the transition of petals from green to white or yellow is accompanied by sharp declines in IAA, ZR, GA, BR, and MeJA, alongside a rise in ABA—a hormonal shift that correlates with chlorophyll loss [8]. Supporting this, exogenous MeJA application accelerates chlorophyll breakdown in *Eustoma* petal, promoting uniform coloration [132]. Deciphering this hormonal control is critical, as it represents a fundamental aspect of floral development with direct application potential. Understanding these mechanisms could enable the directed manipulation of petal color—for instance, by delaying or enhancing degreening to create novel ornamental traits. It could also allow for the modification of floral energy budgets by retaining photosynthetic capacity. Future research could therefore prioritize the systematic mapping of hormonal regulation during petal degreening. While hormones like JA and ABA are implicated in promoting chlorophyll breakdown, their spatiotemporal interactions and the means by which they override signals for chlorophyll biosynthesis or retention are still elusive. Elucidating these pathways will not only resolve a key developmental process but also provide precise tools for manipulating floral coloration and function in breeding programs.

### Transcriptional regulation of chlorophyll accumulation in floral organs

How do key TFs build the core regulatory network linking chlorophyll metabolism to floral organ identity in petals? Addressing this question requires defining the network’s components and functional outputs first. Chlorophyll levels in petals are regulated by several TF families, notably NAC, MYB, CO-like, and TCP [5, 101, 102]. Functional studies of these TFs have demonstrated their impact on pigmentation, as evidenced by the fact that overexpression of *COL16* in petunia increases corolla chlorophyll content [107], TCP loss-of-function in Arabidopsis leads to petal greening [1], and *SnMADS37* in *Solanum nigrum* links petal identity with chlorophyll retention [105]. Deciphering this regulatory network will enable the targeted manipulation of floral color in horticulture and address a key question—how the developmental programs for floral organ identity are integrated with, or uncoupled from, chlorophyll metabolism. Therefore, a major research priority is to move beyond correlative studies and systematically define the core transcriptional circuitry controlling petal chlorophyll metabolism. This requires identification of the master regulatory hubs within key TF families and reconstruction of their hierarchical relationships through integrated functional genomics and comparative analyses across diverse species.

### Floral organ development and chlorophyll accumulation

How can we identify and manipulate the core regulatory nodes that link floral organ identity with chlorophyll metabolism, enabling precise engineering of petal greenness without affecting floral structure or fertility? Answering this first requires understanding how green flowers arise through developmental reprogramming. Some green-flower phenotypes arise from homeotic transformations that convert petals into leaf-like organs [110, 112, 113]. Cultivars such as *Hydrangea* ‘Mini Green,’ *Anthurium andraeanum* Linden, *Dianthus* ‘Green Trick,’ and *Rosa chinensis* cv*.* ‘Viridiflora’ display sepaloid, chlorophyll-containing floral structures (Fig. 5A–D). Such phenotypes are direct manifestations of the intersection between floral organ development and chlorophyll metabolism. The cells within these sepaloid floral structures initiate the program of chloroplast biogenesis and chlorophyll biosynthesis. This indicates that the gene regulatory networks conferring petal identity—particularly those dependent on B/E class genes—are suppressed or altered, whereas the developmental program typically associated with sepals is likely activated ectopically. Evidence suggests that *SnMADS37*, a predicted *SEP3-like* gene in *S. nigrum*, plays a dual role in floral development and chlorophyll metabolism. Its overexpression causes morphological changes and leads to chlorophyll accumulation in petals, correlating with reduced expression of the degradation genes *SnCLH* and *SnPPH*. The mechanism integrating these two functions requires further elucidation [105]. Other studies have established *BANQUO* genes as molecular links between B-class MADS-box activity and chlorophyll accumulation in petals. Mechanistically, petal development is governed by a coherent AP3/PI-mediated repression module that inhibits greening by targeting multiple factors influencing chlorophyll accumulation, including *BNQ* genes. The interaction between factors like GNC/GNL and this BNQ-dependent pathway could represent a valuable direction for future research [119]. The above findings demonstrate that floral identity circuits are closely coupled to chlorophyll regulation—an important consideration for breeders, as altering petal greenness may inadvertently affect flower form and fertility. Understanding this direct link is also critical for both evolutionary biology and applied horticulture. It explains how homeotic transformations produce green flowers in nature and provides the mechanistic knowledge needed to intelligently engineer floral traits.

A key objective for future research is to identify the core regulatory nodes connecting floral organ identity with chlorophyll metabolism. For breeders, this knowledge will enable the development of novel green varieties without the fitness penalties typical of current mutants, by allowing them to decouple pigmentation from morphology. It is crucial to characterize how genes such as *SEP3* homologs and *BNQ* coordinate development and pigmentation, because this will enable the development of strategies that selectively modify petal greenness without compromising normal floral structure or fertility. Employing comparative genomics and targeted genome editing approaches would be particularly valuable in uncovering conserved mechanisms that could be applied across ornamental species.

### Approaches for breeding new green flower varieties in the future

How can we overcome the pleiotropic effects that have hindered the engineering of green flowers, and what integrated strategies (e.g. tissue-specific editing, multi-omics, and fine-tuned expression systems) will enable the precise and stable manipulation of chlorophyll in petals? These precise engineering challenges explain why targeted breeding efforts manipulating chlorophyll metabolism are rare, despite clear commercial potential. Carotenoid and anthocyanin biosynthetic pathways have been extensively manipulated to generate novel floral colors [140, 141], but research on the manipulation of chlorophyll metabolism for green floral color generation remains limited. For ornamental plant breeding, green-flowered mutants are prioritized for selection and development as novel cultivars [142], with green chrysanthemums and carnations already demonstrating horticultural appeal (Fig. 5E and F). However, engineering stable, commercial green flowers requires careful consideration of pleiotropic effects. For instance, overexpression of *LhGLK1* in Arabidopsis induced chlorophyll accumulation in petals but concurrently caused late flowering by interfering with flowering time regulation, revealing its dual role in coordinating chloroplast development and flowering [109]. Similarly, overexpressing *IbMADS3–1* in tobacco led to green petals but also disrupted normal floral development, causing defects in organ identity, pedicel structure, and sepal formation [126]. To achieve precise regulation, floral-promoter-driven strategies such as CRISPR/Cas or RNAi could suppress degradation genes (e.g. *SGR*, *PPH*) or enhance biosynthesis genes exclusively in petals. Future work employing these technologies should carefully balance multiple factors. Multi-omic analyses are needed to identify petal-specific regulatory networks for chlorophyll metabolism, which likely extend beyond leaf ‘stay-green’ mechanisms. Furthermore, multigene strategies and fine-tuned expression systems should balance color modification with plant fitness. Early phenotyping platforms could be crucial to eliminate lines with undesirable side effects.

In summary, green flowers arise from a combination of developmental, metabolic, and environmental factors. Maintaining petal greenness requires deviation from the default maturation program by sustaining chlorophyll biosynthesis, preserving chloroplast identity, and limiting degradation. By integrating developmental genetics, physiology, and evolutionary ecology, future work will not only clarify why green flowers are rare but also open opportunities for breeding novel ornamental traits and deepen our understanding of floral coloration.

## Data Availability

There are no new data associated with this article.
